# A questionnaire-based survey to assess patient satisfaction, ease-of-learning, ease-of-use, injection site pain and overall patient satisfaction of the follitropin-alpha (Gonal-f) filled-by-mass (FbM) prefilled pen compared with other systems of gonadotrophin administration

**DOI:** 10.1186/1477-7827-10-93

**Published:** 2012-11-20

**Authors:** Takafumi Utsunomiya, Atsushi Tanaka, Kenichi Tatsumi, Diego Ezcurra

**Affiliations:** 1 Japanese Institution for Standardizing Assisted Reproductive Technology, Osaka, Japan; 2 Fertility and Endocrinology Business Unit, Merck Serono SA, Geneva, Switzerland

**Keywords:** Self-administration, Injections, Fertility agents, Follicle-stimulating hormone, Ovulation induction, Assisted reproductive techniques, Patient satisfaction

## Abstract

**Background:**

Gonadotrophins are used routinely for follicular stimulation during ovarian induction and assisted reproduction techniques. Developments in recombinant follicle-stimulating hormone preparations and their injection devices have improved patient quality of life by enabling patients to self-administer treatment at home. The objective of this study was to investigate patient experiences of learning to use and overall satisfaction with the follitropin-alpha (Gonal-f) filled-by-mass (FbM) prefilled pen.

**Methods:**

This questionnaire-based survey study was conducted in 23 fertility centres in Japan over a period of 14 months. Patients who were receiving fertility treatment with the follitropin-alpha (FbM) prefilled pen were asked to complete a questionnaire to assess their satisfaction, ease of learning and use, and injection site pain following treatment.

**Results:**

A total of 663 women participated in the study. The majority of patients found the instructions for administering follitropin-alpha with the prefilled pen easy to understand (83.0%; n = 546/658) and patients found that a hands-on demonstration by a nurse or doctor was the most useful tool for learning to use the follitropin-alpha (FbM) prefilled pen (80.0%; n = 497/621). Forty-eight percent (n = 318) of patients in the study had previous experience with different types of fertility medications and the majority of these patients found the follitropin-alpha (FbM) prefilled pen easier to use (75.1%; n = 232/309) and less painful (89.0%; n = 347/390) than their previous medication. The majority (80.2%; n = 521/650) of patients reported overall satisfaction with the follitropin-alpha (FbM) prefilled pen.

**Conclusions:**

The follitropin-alpha (FbM) prefilled pen is an easy-to-use injection device according to this questionnaire-based survey. Patients who had experience of different types of fertility medication preferred the follitropin-alpha (FbM) prefilled pen to other injection devices.

## Background

Gonadotrophins are routinely used for follicular stimulation in ovarian induction (OI) and assisted reproduction techniques (ART). Follicle-stimulating hormone (FSH) and HMG (human menopausal gonadotrophin) are used during OI and ART to stimulate the growth and recruitment of immature follicles in the ovary [1,2]. FSH is currently available in two preparations: urinary FSH (u-FSH) and recombinant FSH (r-FSH). HMG and u-FSH are extracted from the urine of postmenopausal women, whereas r-FSH, which became available in 1996, is produced using recombinant DNA technology.

Daily injections of recombinant peptides are required for several conditions such as growth hormone for growth deficiencies in children. Injections that are required on a long-term, regular basis must be quick, simple and as pain free as possible to ensure patients comply with treatment [3]. During treatment for OI and ART, gonadotrophins have to be injected daily for an average of 10 days and are often reconstituted from freeze-dried peptides. Traditionally, HMG has been administered as an intramuscular injection. However, the development of the higher purity FSH preparations has enabled subcutaneous injections to be used. Advances in FSH formulations have been accompanied by progress in the devices available for the delivery of the peptides. The subcutaneous administration systems that have evolved for r-FSH generally enable self-administration at home and have led to improvements in patient satisfaction and quality of life [4-10].

It is well established that treatment outcomes can be affected by patient compliance, and that compliance is related to treatment choice and method of administration. For example, therapies that are easier to administer and cause less injection site pain are associated with higher rates of compliance than those that are difficult to administer and cause pain [4,7,10,11]. It has been demonstrated previously that patients, who receive subcutaneous injections for IVF, reported experiencing less pain than those who receive intramuscular injections [12]. Moreover, it has been documented that patients can make errors in the reconstitution of freeze-dried peptides for self-administration at home, and pregnancy rates are higher in patients who prepare and administer injections correctly [10].

The follitropin-alpha (Gonal-f) filled-by mass (FbM) prefilled pen (Merck Serono SA, Geneva, Switzerland, an affiliate of Merck KGaA, Darmstadt, Germany) is a ready-to-use disposable injection delivery device. The pen contains premixed follitropin alfa filled-by-mass, which provides more accurate and consistent dosing than r-FSH filled-by-bioassay [8]. Routine use of follitropin-alpha in ART was well accepted by patients participating in a German observational study; in addition, study patients required less training to use follitropin-alpha pens compared with vials or ampoules [13]. More importantly from a compliance point of view, patients preferred the prefilled pen compared with another loadable device (a reusable pen with loadable cartridges) [13]. Results from two other studies suggest that the follitropin-alpha prefilled pen had higher patient and nurse acceptance than the follitropin-beta pen (supplied in glass cartridges to be used with a pen injector; Puregon; Organon, Roseland, NJ, USA) [14]. Another survey found that more patients preferred the follitropin-alpha prefilled pen to the follitropin-beta cartridge pen or urofollitropin (Ferring Pharmaceuticals, Suffern, NY, USA) administered with a needle-free reconstitution device and conventional syringe. Additionally, patients stated that the ease of use, dosing mechanism and reduced potential for dosing errors were the factors that they found most important [15].

The current study was designed to evaluate overall patient satisfaction, ease of learning and use, and injection site pain associated with the follitropin-alpha (FbM) prefilled pen. A secondary comparison was made with other systems of gonadotrophin administration. The study—which involved over 650 patients treated at 23 Japanese fertility centres—was the largest study of patient preference to be performed in Japan.

## Methods

### Study design

This was a questionnaire-based survey conducted in 23 clinics certified by the Japanese Institution for Standardising Assisted Reproductive Technology (JISART). JISART aims to achieve high standards of practice in fertility treatments by implementing a quality management system, with the ultimate goal of improving the quality of patient care.

### Patients

Women receiving OI or ART fertility treatment with the follitropin-alpha (FbM) prefilled pen in their current treatment cycle were eligible for inclusion in the study. No further inclusion or exclusion criteria were used. Patients from 23 JISART-certified clinics were recruited into the study over 14 months from October 2009 to December 2010, with a target recruitment number of 650.

### Assessment

Patients undergoing treatment with the follitropin-alpha (FbM) prefilled pen were asked to complete a questionnaire to assess their ease of learning and use, injection site pain and overall satisfaction. The first part of the questionnaire was completed after appropriate instruction was given for the patients to learn how to use the device and included 7 multiple-choice questions that were associated with how easy patients found it to learn to use the follitropin-alpha (FbM) prefilled pen. The second part of the questionnaire, completed at the end of the treatment, consisted of 10 questions that assessed how easy patients found the pen to use, how painful patients found the pen and the patients’ overall satisfaction with the pen. If a patient had experience in previous treatment cycles of using formulations of FSH other than follitropin-alpha, with different methods of administration, she was asked to compare the follitropin-alpha (FbM) prefilled pen with the previous therapy. This section of the questionnaire enabled comparison of the follitropin-alpha (FbM) prefilled pen with other systems of gonadotrophin administration. The scoring system for the questionnaire ranged from 1 to 10 for ease of learning and use, as well as overall satisfaction with a score of 10 reflecting the most positive experience. The scoring for injection site pain also ranged from 1 to 10 on a numerical rating scale, with 10 being the most painful experience.

### Reporting methods

Patient responses were calculated as a percentage of the population who responded to each question. Studies that use patient questionnaires to gather information invariably have some data missing from the completed forms. In these cases, the responses were not included in the final analysis.

## Results

### Patient and treatment characteristics

A total of 663 women who were prescribed follitropin-alpha (FbM) prefilled pen (OI = 39.4%, n = 261/663; ART = 60.6%, n = 402/663) at 23 JISART-certified clinics were recruited into the study. The majority of participating women self-administered their injections (68.5%; n = 454/663) or partly self-administered their injections (27.9%; n = 185/663).

### Ease of learning

A total of 663 women who were receiving treatment with the follitropin-alpha (FbM) prefilled pen in their current treatment cycle participated in the questionnaire-based study. Patients were asked a series of questions to assess how easy it was to learn to use the follitropin-alpha (FbM) prefilled pen and a full list of questions and tallied responses are shown in Table 1.

**Table 1:** Ease of learning*

**Question**	**Possible answers**		**Patient response (n)**	**Patient response (%)**	**Total no. patients who responded to question**
Were the instructions for administering the follitropin-alpha pen easy to understand?	Yes		546	83.0	658
	OK		106	16.1	658
	No		6	0.9	658
	N/A		5		
Which tools did you use to learn how to use the follitropin-alpha pen and how useful were they?	Hands-on demonstration by nurse/doctor	Very useful	582	91.5	636
		Useful	52	8.2	636
		Not useful	2	0.3	636
		N/A	27		
	Leaflet	Very useful	421	71.1	592
		Useful	161	27.2	592
		Not useful	10	1.7	592
		N/A	71		
	DVD	Very useful	160	47.3	338
		Useful	135	39.9	338
		Not useful	43	12.7	338
		N/A	325		
	Web	Very useful	18	26.1	69
		Useful	21	30.4	69
		Not useful	30	43.5	69
		N/A	594		
Which of the tools did you find was the most useful in learning how to use the follitropin-alpha pen?	Hands-on demonstration by nurse/doctor		497	80.0	621
	Leaflet		89	14.3	621
	DVD		35	5.6	621
	Web		0	0.0	621
	N/A		42		
How easy was it for you to understand how to use the follitropin-alpha pen?	1 (very difficult)		3	0.5	658
	2		4	0.6	658
	3		11	1.7	658
	4		5	0.8	658
	5		26	4.0	658
	6		21	3.2	658
	7		49	7.4	658
	8		152	23.1	658
	9		128	19.5	658
	10 (very easy)		259	39.4	658
	N/A		5		
Based on your experience of learning, would you ecommend the follitropin-alpha pen to another woman who was considering fertility treatment?	Yes		611	94.9	644
	No		33	5.1	644
	N/A		19		
Have you used any of the following medications in the past? If yes, please select the medication that you most recently used^†^	Follistim pen		130	40.9	318
	Follistim vial		53	16.7	318
	hMG		119	37.4	318
	Gonapure		6	1.9	318
	Folyrmon P		10	3.1	318
	N/A or no previous use		348		
Which treatment did you find to be the easiest to use: the follitropin-alpha pen or a previous treatment medication?	Follitropin-alpha pen		232	75.1	309
	Same ease of use		70	22.7	309
	Other product		7	2.3	309
	N/A or no use		354		

*Patients were asked a series of questions to assess how easy it was to learn to use the follitropin-alpha (FbM) prefilled pen. N/A = patient did not answer the question. ^†^ Includes multiple answers.

The majority of patients surveyed (83.0%; n = 546/658) found the instructions for administering follitropin-alpha with the prefilled pen easy to understand and patients gave an average score of 8.54 out of 10 for ease of learning (1 = difficult to understand; 10 = easy to understand). Several tools were employed to teach patients how to use the follitropin-alpha (FbM) prefilled pen, including doctor/nurse’s guidance, a printed leaflet, a DVD video and a website page. Women found doctor/nurse’s guidance (91.5%; n = 582/636), a printed leaflet (71.1%; n = 421/592) and DVD (47.3%; n = 160/338) were the most effective learning tools, whereas the majority of patients did not use, or did not confirm whether they had used, the website page as a training aid (89.6%; n = 594/663) or did not find it useful (43.5%; n = 30/69). Overall, 80.0% (n = 497/621) of patients found that a hands-on demonstration by a nurse or doctor was the most useful training tool, 14.3% (n = 89/621) found a printed leaflet was the most useful tool and 5.6% (n = 35/621) found a DVD the most useful, whereas no women found the website page to be the most useful tool.

Forty-eight percent of patients in the study had previous experience with gonadotropin medications, other than follitropin-alpha, for the treatment of infertility. These were the follitropin-beta pen (rFSH, Follistim cartridge-type pen; Organon, Roseland, NJ, USA; 40.9%; n = 130/318), hMG (vial, ampoule and syringe; 37.4%; n = 119/318), follitropin-beta vial (rFSH, Follistim vial; Organon, Roseland, NJ, USA; 16.7%; n = 53/318), Folyrmon P (uFSH, ampoule and syringe; Fuji Seiyaku Kogyo, Chuo-ku, Tokyo, Japan; 3.1%; n = 10/318) and Gonapure (uFSH, ampoule and syringe, ASKA Pharmaceutical, Tokyo, Japan; 1.9%; n = 6/318). When questioned if they found the follitropin-alpha (FbM) prefilled pen easier to learn to use than a previous treatment, 75.1% (n = 232/309) of patients stated that follitropin-alpha was easier to use, 22.7% (n = 70/309) thought there was no difference in ease of use between treatments and 2.3% (n = 7/309) believed their previous treatment was easier to use. When patients were questioned as to whether they would recommend the follitropin-alpha (FbM) prefilled pen to another woman considering fertility treatment based on how easy they found it to learn to use, the majority of patients said they would recommend follitropin-alpha (94.9%; n = 611/644).

### Ease of use and injection site pain

**Figure 1:**
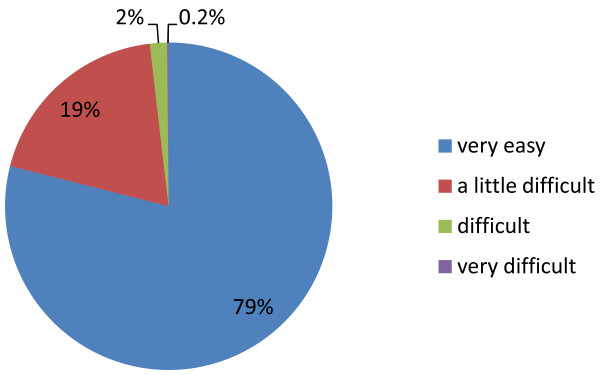
**Ease of use.** Pie chart shows the percentage of patients responding by category to the question ‘How easy was it for you to use the follitropin-alpha pen?’ (n=663).

Patients were also asked a series of questions to assess how easy they found the follitropin-alpha (FbM) prefilled pen to use and their overall satisfaction with the device (Table 2**)**. Patients gave an average score of 8.65 out of 10 for ease of use (1 = difficult to use; 10 = easy to use). Almost all of the patients responded that the follitropin-alpha (FbM) prefilled pen was very easy to use (78.9%; n = 514/651) or that they had little difficulty in self-injection (19.2%; n = 125/651) (Figure 1), and 99.1% (n = 639/645) of the women were able to administer some or all of their own injections with the follitropin-alpha (FbM) prefilled pen. Seventy-five percent (232/309) of women who had prior experience of other treatments for infertility found injection with the follitropin-alpha (FbM) prefilled pen easier to learn to use than the prior therapy.

**Table 2:** Ease of use and level of satisfaction*

**Question**	**Possible answers**	**Patient response (n)**	**Patient response (%)**	**Total no. patients who responded to question**
How easy did you find the self-injection of the follitropin-alpha pen?	Very easy	514	79.0	651
	A little difficult	125	19.2	651
	Difficult	11	1.7	651
	Very difficult	1	0.2	651
	N/A	12		
How easy was it for you to use the follitropin-alpha pen?	1 ( very difficult)	5	0.8	651
	2	4	0.6	651
	3	7	1.1	651
	4	8	1.2	651
	5	24	3.7	651
	6	7	1.1	651
	7	41	6.3	651
	8	129	19.8	651
	9	174	26.7	651
	10 (very easy)	252	38.7	651
Did you administer the injections yourself during this treatment cycle?	Yes, completely	454	70.4	645
	Yes, some injections	185	28.7	645
	No	6	0.9	645
	N/A	18		
Did you use the follitropin-alpha pen (self-injection) outside the home?	Yes, I have injected outside the home	116	17.8	651
	No, all injections were administered at home	535	82.2	651
	N/A	12		
	*If Yes, how convenient was it to carry the follitropin-alpha pen with you?*			
	Very good	49	43.0	114
	Good	60	52.6	114
	Bad	5	4.4	114
How confident did you feel that you accurately administered your daily dose using the follitropin-alpha pen during this treatment?	Very confident	283	43.8	646
	Somewhat confident	357	55.3	646
	Not at all confident	6	0.9	646
	N/A	17		
How painful was the injection with the follitropin-alpha pen	0 (no pain)	139	21.2	657
	1	236	35.9	657
	2	123	18.7	657
	3	77	11.7	657
	4	21	3.2	657
	5	36	5.5	657
	6	13	2.0	657
	7	5	0.8	657
	8	4	0.6	657
	9	2	0.3	657
	10 (most pain)	1	0.2	657
Which was less painful; follitropin-alpha pen or other product?	Follitropin-alpha pen	347	89.0	390
	Same	39	10.0	390
	Other product	4	1.0	390
	N/A or No use of other product	273		
Overall, how satisfied are you with the follitropin-alpha pen	Satisfied	521	80.2	650
	Neither satisfies or not satisfied	124	19.1	650
	Not satisfied	5	0.8	650
	N/A	13		
How would you rate the overall level of satisfaction when using the follitropin-alpha pen?	1 (not satisfied at all)	4	0.6	651
	2	2	0.3	651
	3	4	0.6	651
	4	5	0.8	651
	5	34	5.2	651
	6	18	2.8	651
	7	42	6.5	651
	8	141	21.7	651
	9	153	23.5	651
	10 (very satisfied)	248	38.1	651
Based on your experience, would you recommend follitropin-alpha pen to another woman considering fertility treatment?	Yes	596	93.9	635
	No	39	6.1	635
	N/A	28		

*Patients were asked a series of questions to assess how easy it was to use the follitropin-alpha (FbM) prefilled pen and to assess overall levels of satisfaction with the pen. N/A = patient did not answer the question.

**Figure 2:**
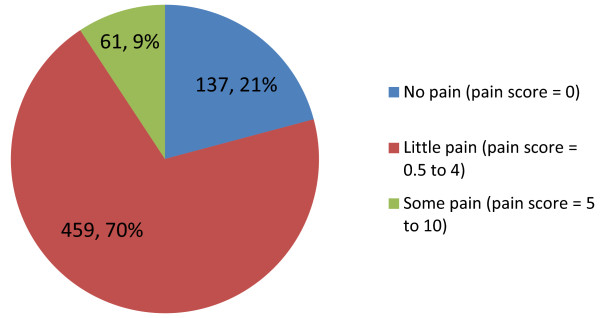
**Injection site pain during use.** Pie chart shows the percentage of patients responding by category to the question ‘How painful was the injection with the Gonal-f pen?’ (n=663).

When questioned how painful they found injection with the follitropin-alpha (FbM) prefilled pen, almost a quarter of respondents said they experienced no pain (score of 0; 20.9%; n = 137/657) and a little pain was reported by the majority of patients (score of between 0.5 and 4; 69.9%; n = 459/657) (Figure 2). Among the patients who had prior experience of other treatments for infertility, 89.0% (n = 347/390) found follitropin-alpha to be less painful to use than the other treatment.

### Overall patient satisfaction

**Figure 3:**
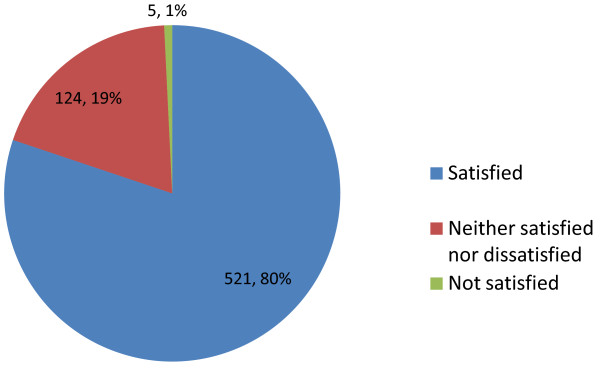
**Level of patient satisfaction.** Pie chart shows the percentage of patients responding by category to the question ‘How would you rate the overall level of satisfaction when using the follitropin-alpha pen?’ (n=663).

**Figure 4:**
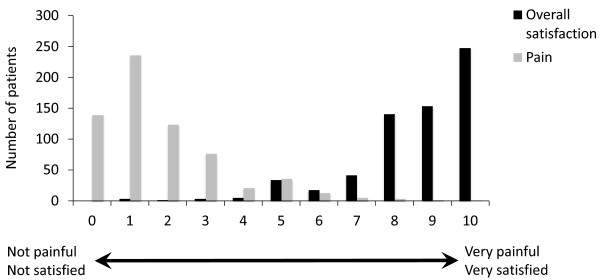
**Ease of learning, ease of use and overall satisfaction.** Patients were asked to rate the ease of learning, ease of use and overall satisfaction with the follitropin-alpha pen on a scale of 1 to 10 (n = 663).

**Figure 5:**
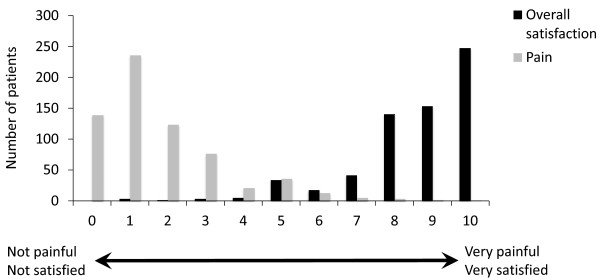
**Injection site pain and overall satisfaction.** Patients were asked to rate the injection site pain and overall satisfaction with the follitropin-alpha (FbM) pen on a scale of 1 to 10 (n = 663).

The majority (80.2%; n = 521/650) of women reported being satisfied with their treatment experience using the follitropin-alpha (FbM) prefilled pen compared with 19.1% (n = 124/650) who were neither satisfied nor dissatisfied and 0.7% (n = 5/650) who were not satisfied (Figure 3). The follitropin-alpha (FbM) prefilled pen received an average score of 8.6 out of 10 (1 = not satisfied; 10 = very satisfied). Nearly all (93.9%; n = 596/635) of survey respondents said they would recommend using the follitropin-alpha (FbM) prefilled pen to another woman considering fertility treatment. Patients who found the follitropin-alpha (FbM) prefilled pen easy to learn and use demonstrated the highest overall satisfaction (Figure 4). However, there was no direct correlation between ease of learning and use, and overall satisfaction. Additionally, patients who experienced little pain at the injection site when using the follitropin-alpha (FbM) prefilled pen had higher overall satisfaction than patients who experienced pain at the injection site (Figure 5).

## Discussion

The results from this questionnaire-based survey—which involved 663 women and was the largest of its type to be performed in Japan—show that the majority of patients found the follitropin-alpha pen easy to learn, to use and to administer with minimal pain experienced at the injection site. Higher overall patient satisfaction appeared to be associated with higher scores for ease of learning and use, as well as less pain at the injection site, although no direct correlation between these factors was found. Seventy-five percent (232/309) of patients who had experience of other treatments for infertility found the follitropin-alpha (FbM) prefilled pen the easiest product to use and almost all would recommend the use of the follitropin-alpha pen to other women. These results are comparable to those from two other studies reported in a single publication by Porter et al. [14]. The first was a 2-year observational study conducted in Germany, which found that patients favoured treatment with the follitropin-alpha (FbM) prefilled pen over the follitropin-beta pen due to faster preparation, greater confidence in dosing accuracy and the need for fewer dose adjustments [14]. In the second study, conducted in Australia, patients again favoured the follitropin-alpha (FbM) prefilled pen over the follitropin-beta pen for the same reasons. Additionally, patients experienced less injection site pain with the follitropin-alpha (FbM) prefilled pen than with the follitropin-beta pen [14]. A larger questionnaire-based study of 5328 patients undergoing ART in Germany reported that the routine use of the follitropin-alpha (FbM) prefilled pen was well accepted by patients [13]. In this study, patients found the follitropin-alpha (FbM) prefilled pen easier to learn to use than other injection methods and reported it as their preferred method of FSH administration [13].

The current study included an exploration of patients’ views on the different tools provided to teach them how to use the follitropin-alpha (FbM) prefilled pen. Surprisingly, web-based training was not widely utilised and was associated with lower overall satisfaction than the other training tools. Patients found hands-on demonstration by a doctor or nurse to be the most useful tool for learning how to use the follitropin-alpha (FbM) prefilled pen. When a healthcare professional demonstrates how to perform the injection, patients have the chance to ask any questions they may have on correct administration and what they should do if they encounter any problems. Asking a doctor or nurse can provide patients with the reassurance that they need to correctly administer the treatment at home by themselves. Interestingly, nurses have also reported very high levels of satisfaction with their experiences of teaching patients to use the follitropin-alpha pen [14].

One of the advantages of a ready-to-use device is that it reduces the chances of a reconstitution error occurring. The follitropin-alpha (FbM) prefilled pen lowers the possibility of both injection and dosing errors, which could potentially affect patient confidence in the device as well as the choice of device used in fertility programmes. Additionally, increased patient confidence, dosing consistency and dosing accuracy are key requirements for a drug delivery device to allow physicians to maximise the predictability of ovarian stimulation and success [10].

When questioned, over three quarters of the patients in the study who had previous experience of other gonadotrophin formulations and devices found the follitropin-alpha pen easier to use and reported less injection site pain than with other products. Of note, in a study of patients receiving human growth hormone injections, the five key delivery device attributes identified by patients were reliability, ease of use, lack of pain, safety in use and a small number of steps required for preparation [16]. The follitropin-alpha (FbM) prefilled pen encompasses all of these attributes. Also worthy of consideration is that the introduction of insulin self-injection pens for patients with diabetes led to an improvement in patient compliance and treatment outcomes, with patients reporting that they found injection pens less painful than syringes and needles [17-22]. It is likely that the use of injection pens for infertility treatment that cause less injection site pain than conventional syringes will have a similar positive effect on compliance. It has also been demonstrated in many therapeutic areas that increasing patient information leads to increased patient satisfaction and adherence to treatment [23-26].

Additionally, it has been suggested that the efficacy of ovarian stimulation regimens may improve with the use of injection pen devices compared with conventional syringes. This could be due to several factors such as increased adherence to treatment and also the correct administration and dosing of treatment. However, further evidence is required to support these claims [27-29].

In summary, the follitropin-alpha (FbM) prefilled pen is an easy-to-use injection device and offers reduced injection site pain compared with conventional devices. A high level of overall patient satisfaction with the follitropin-alpha (FbM) prefilled pen was documented in this study involving 23 JISART-certified fertility centres in Japan. The results of this survey are in agreement with previous findings confirming the ease of learning and use of the pen in both previously untreated patients and patients with previous experience of other fertility treatments.

## Abbreviations

ART: Assisted reproduction technique; FbM: Filled-by-mass; FSH: Follicle-stimulating hormone; HMG: Human menopausal gonadotrophin; JISTART: Japanese Institution for Standardising Assisted Reproductive Technology; OI: Ovarian induction; r-FSH: Recombinant follicle-stimulating hormone; u-FSH: Urinary follicle-stimulating hormone.

## Competing interests

Diego Ezcurra is an employee of Merck Serono SA, Geneva, Switzerland (an affiliate of Merck KGaA, Darmstadt, Germany). Other authors have no conflicting interests.

## Authors’ contributions

TU contributed to the conduct, and analysis of the study. AT contributed to the conception, conduct and overall coordination of the multi-centre study. KT contributed to the conduct of the study and DE contributed to the conception and analysis of the study. All authors read and approved the final manuscript.

## References

[B1] Oehninger S Ovulation induction in IVF Minerva Ginecol 2011 63 137 156 21508903

[B2] Oehninger S Hodgen GD Induction of ovulation for assisted reproduction programmes Baillieres Clin Obstet Gynaecol 1990 4 541 573 10.1016/S0950-3552(05)80310-4 2282742

[B3] Mahato RI Narang AS Thoma L Miller DD Emerging trends in oral delivery of peptide and protein drugs Crit Rev Ther Drug Carrier Syst 2003 20 153 214 10.1615/CritRevTherDrugCarrierSyst.v20.i23.30 14584523

[B4] Pang SC A pen injection device for self-administration of recombinant follicle-stimulating hormone for fertility treatments Expert Rev Med Devices 2005 2 27 32 10.1586/17434440.2.1.27 16293025

[B5] Somkuti SG Schertz JC Moore M Ferrande L Kelly E Patient experience with follitropin alfa prefilled pen versus previously used injectable gonadotropins for ovulation induction in oligoanovulatory women Curr Med Res Opin 2006 22 1981 1996 10.1185/030079906X132604 17022858

[B6] Platteau P Laurent E Albano C Osmanagaoglu K Vernaeve V Tournaye H Camus M Van Steirteghem A Devroey P An open, randomized single-centre study to compare the efficacy and convenience of follitropin beta administered by a pen device with follitropin alpha administered by a conventional syringe in women undergoing ovarian stimulation for IVF/ICSI Hum Reprod 2003 18 1200 1204 10.1093/humrep/deg234 12773446

[B7] Sedbon E Wainer R Perves C Quality of life of patients undergoing ovarian stimulation with injectable drugs in relation to medical practice in France Reprod Biomed Online 2006 12 298 303 10.1016/S1472-6483(10)61001-2 16569316

[B8] Bassett RM Driebergen R Continued improvements in the quality and consistency of follitropin alfa, recombinant human FSH Reprod Biomed Online 2005 10 169 177 10.1016/S1472-6483(10)60937-6 15823219

[B9] Craenmehr E Bontje PM Hoomans E Voortman G Mannaerts BM Follitropin-beta administered by pen device has superior local tolerance compared with follitropin-alpha administered by conventional syringe Reprod Biomed Online 2001 3 185 189 10.1016/S1472-6483(10)62033-0 12513852

[B10] Markle RL King PJ Martin DB Characteristics of successful human chorionic gonadotrophin (hCG) administration in assisted reproduction [abstract] Fertil Steril 2002 78 Suppl 1 S71 S72

[B11] Pang S Kaplan B Karande V Westphal LM Scott R Givens C Sacks P Administration of recombinant human FSH (solution in cartridge) with a pen device in women undergoing ovarian stimulation Reprod Biomed Online 2003 7 319 326 10.1016/S1472-6483(10)61871-8 14653893

[B12] Alviggi C Revelli A Anserini P Ranieri A Fedele L Strina I Massobrio M Ragni N De PG A prospective, randomised, controlled clinical study on the assessment of tolerability and of clinical efficacy of Merional (hMG-IBSA) administered subcutaneously versus Merional administered intramuscularly in women undergoing multifollicular ovarian stimulation in an ART programme (IVF) Reprod Biol Endocrinol 2007 5 45 10.1186/1477-7827-5-45 18053198 PMC2216030

[B13] Weckler Post marketing surveillance analysis of the routine use of follitrophin alpha in a prefilled ready to use injection pen in ART [abstract] Fertil Steril 2006 86 S411

[B14] Porter R Kissel C Saunders H Keck C Patient and nurse evaluation of recombinant human follicle-stimulating hormone administration methods: comparison of two follitropin injection pens Curr Med Res Opin 2008 24 727 735 10.1185/030079908X273291 18230195

[B15] Weiss N Gonadotrophin products: empowering patients to choose the product that meets their needs Reprod Biomed Online 2007 15 31 37 10.1016/S1472-6483(10)60688-8 17623531

[B16] Dumas H Panayiotopoulos P Parker D Pongpairochana V Understanding and meeting the needs of those using growth hormone injection devices BMC Endocr Disord 2006 6 5 10.1186/1472-6823-6-5 17034628 PMC1618831

[B17] Bohannon NJ Insulin delivery using pen devices. Simple-to-use tools may help young and old alike Postgrad Med 1999 106 57 68 10560468 10.3810/pgm.1999.10.15.751

[B18] Brunton S Initiating insulin therapy in type 2 diabetes: benefits of insulin analogs and insulin pens Diabetes Technol Ther 2008 10 247 256 10.1089/dia.2008.0287 18715198

[B19] Hanestad BR Albrektsen G Quality of life, perceived difficulties in adherence to a diabetes regimen, and blood glucose control Diabet Med 1991 8 759 764 10.1111/j.1464-5491.1991.tb01696.x 1838068

[B20] Pfutzner A Asakura T Sommavilla B Lee W Insulin delivery with FlexPen: dose accuracy, patient preference and adherence Expert Opin Drug Deliv 2008 5 915 925 10.1517/17425247.5.8.915 18713000

[B21] Kadiri A Chraibi A Marouan F Ababou MR el Guermai N Wadjinny A Kerfati A Douiri M Bensouda JD Belkhadir J Comparison of NovoPen 3 and syringes/vials in the acceptance of insulin therapy in NIDDM patients with secondary failure to oral hypoglycaemic agents Diabetes Res Clin Pract 1998 41 15 23 10.1016/S0168-8227(98)00055-2 9768368

[B22] Sucic M Galic E Cabrijan T Ivandic A Petrusic A Wyatt J Mincheva N Milicevic Z Malone J Patient acceptance and reliability of new Humulin/Humalog 3.0 ml prefilled insulin pen in ten Croatian diabetes centres Med Sci Monit 2002 8 I21 I26 11884952

[B23] Heisler M Bouknight RR Hayward RA Smith DM Kerr EA The relative importance of physician communication, participatory decision making, and patient understanding in diabetes self-management J Gen Intern Med 2002 17 243 252 10.1046/j.1525-1497.2002.10905.x 11972720 PMC1495033

[B24] Schattner A Bronstein A Jellin N Information and shared decision-making are top patients’ priorities BMC Health Serv Res 2006 6 21 10.1186/1472-6963-6-21 16507096 PMC1431526

[B25] Hovatta O McVeigh E Lass A Homburg R A large Northern European observational study of follitropin alpha filled-by-mass pre-filled pen Reprod Biomed Online 2009 18 502 508 10.1016/S1472-6483(10)60126-5 19400991

[B26] DiMatteo MR The physician-patient relationship: effects on the quality of health care Clin Obstet Gynecol 1994 37 149 161 10.1097/00003081-199403000-00019 8194205

[B27] Chen LN Quan S Li H Yang XP Chen SM Zhang XY Lin L Xing FQ Chen SL Wan ZJ Clinical application of prefilled pen and conventional syringe during controlled ovarian stimulation for in vitro fertilization Nan Fang Yi Ke Da Xue Xue Bao 2009 29 100 104 19218125

[B28] Christianson MS Barker MA Schouweiler C Lindheim SR A retrospective comparison of clinical outcomes and satisfaction using reconstituted recombinant gonadotropins (rFSH) or cartridge rFSH with a pen device in donor oocyte cycles Curr Med Res Opin 2007 23 865 870 10.1185/030079907X178784 17407643

[B29] Rama Raju GA Suryanarayana K Jaya PG Murali KK Comparison of follitropin-beta administered by a pen device with conventional syringe in an ART programme - a retrospective study J Clin Pharm Ther 2008 33 401 407 10.1111/j.1365-2710.2008.00931.x 18613858

